# Supplementary material for: Decreased thermal niche breadth as a trade-off of antibiotic resistance
Source: ISME J. 2022 Apr 14;16(7):1843–52. doi: 10.1038/s41396-022-01235-6 (PMC9213455; doi:10.1038/s41396-022-01235-6)
Supplement: Supplementary file 1 — Supplementary Online Methods [file 41396_2022_1235_MOESM1_ESM.docx]

**Supplemental Materials:**

These figures show in detail the data processing pipeline (Supplementary Figure 1) and the robustness of the results to changes in the data processing parameters (Supplementary Figure 2).

**Supplementary Figure 1:** Panels show the progression from raw data (left-hand panel) to calculated maximum growth rates (right-hand panel). The left-most plot shows two examples of raw growth curves from the plate reader, which gives optical density over time in 5-minute intervals. The second panel shows these data where each curve is centered to have a minimum value of 0.02. This adjustment removes the minor variation in initial values by well, and also takes out the background optical density level that originates from the media and plate lid. Next, a local linear model is used to smooth the log values. The number of points used to create the linear model is referred to as the width of the model, which was 24 in the main text. Finally, taking the first difference of sequential values yields the right-most panel. The maximum growth rates (dashed lines) are measured as the largest difference between sequential, log-transformed, smoothed optical density values.

Minimum Value: 0.01; Smoothing Width: 18 Minimum Value: .01; Smoothing Width: 30

Minimum Value 0.03; Smoothing Width: 18 Minimum Value: 0.03; Smoothing Width: 30

**Supplementary Figure 2:** Results shown in the main text are robust to using alternate values in the data processing. The four sets of figures show the same data presented in Fig. 4, but use different values for either the minimum optical density value (either 0.01 or 0.03, instead of 0.02) and the smoothing width (either 18 or 30, instead of 24). Changing the minimum value alters the magnitude of the growth rates, as the log-transformed differences are larger when the minimum value is 0.01 and smaller when it is 0.03. However, the relative differences are largely preserved. Changing the smoothing to have a larger width decreases the calculated growth rates at 42C, perhaps because the period of rapid growth is shorter in these cultures. However, again, the qualitative results are insensitive to the number of points used for smoothing.
