# Supplementary material for: Decreased thermal niche breadth as a trade-off of antibiotic resistance
Source: ISME J. 2022 Apr 14;16(7):1843–52. doi: 10.1038/s41396-022-01235-6 (PMC9213455; doi:10.1038/s41396-022-01235-6)
Supplement: Supplementary file 2 — Data Analysis Scripts [file 41396_2022_1235_MOESM2_ESM.docx]

## Analyze merged flow cytometry data

library(lme4)

library(RColorBrewer)

library(ggplot2)

library(dplyr)

library(gridExtra)

library(nlme)

all.ev <- read.csv("~/Desktop/MergedFlowCytometryDataset.csv")

# Relevel temperatures so linear model compares against 37 as baseline

all.ev$temp <- as.factor(all.ev$temp)

all.ev$temp <- relevel(all.ev$temp, ref = "37")

# Conduct analyses of log-ratios

summary(lme(log(gfp.rawratio) ~ -1 + lineage * gfp.timediff * temp , random = ~ 1|mchstrain, data = all.ev) )

## Count which lineages have interaction:

## 1, 2, 7, 10, 11, 13, 15, 18, 19, 20, 21, 22

# Collapse all temperature interactions by lineage to see overall effect of temperature

summary(lme(log(gfp.rawratio) ~ lineage * temp + gfp.timediff * temp , random = ~ 1|mchstrain, data = all.ev) )

anova(lme(log(gfp.rawratio) ~ lineage * temp + gfp.timediff * temp , random = ~ 1|mchstrain, data = all.ev) )

#Make plots of same lineage at different temperatures

par(mfrow = c(1, 2))

lineage.plot1 <- "L5"

plot(all.ev$gfp.rawratio[all.ev$lineage == lineage.plot1] ~ all.ev$gfp.timediff[ all.ev$lineage == lineage.plot1], xlab = "Resistance Difference of Strains", ylab = "Ratio of Performance of GFP Strain", log = "y", pch = 20, main = "Lineage 5", col = rep(c("blue", "black", "red"), each = 48) )

legend("bottomleft", bty = "l", legend = c("32", "37", "42"), col = c("blue", "black", "red"), pch = 20)

lineage.plot2 <- "L19"

plot(all.ev$gfp.rawratio[all.ev$lineage == lineage.plot2] ~ all.ev$gfp.timediff[ all.ev$lineage == lineage.plot2], xlab = "Resistance Difference of Strains", ylab = "Ratio of Performance of GFP Strain", log = "y", pch = 20, main = "Lineage 19", col = rep(c("blue", "black", "red"), each = 48) )

## Next, make heatmaps of gfp ratios at the 3 temperatures. Should be 4x4 grid of GFP and mCh concentrations

# First split up tapply names to get resistance levels

all.ev$combo_temp <- paste(all.ev$gfptime, all.ev$mchtime, all.ev$temp, sep = "_")

name.split <- as.data.frame(strsplit(names(tapply(all.ev$gfp.rawratio, all.ev$combo_temp, mean)), "_"))

name.split <- rbind(name.split, tapply(all.ev$gfp.rawratio, all.ev$combo_temp, median) )

name.split <- t(name.split)

colnames(name.split) <- c("gfp.time", "mch.time", "temp", "ratio")

name.split <- as.data.frame.matrix(name.split)

name.split$log.ratio <- log(as.numeric(name.split$ratio))

name.split32 <- name.split[name.split$temp == "32", ]

name.split37 <- name.split[name.split$temp == "37", ]

name.split42 <- name.split[name.split$temp == "42", ]

gfp.levels <- as.data.frame(strsplit(names(tapply(all.ev$gfp.rawratio, all.ev$combo_temp, mean)), "_"))[1, ]

mch.levels <- as.data.frame(strsplit(names(tapply(all.ev$gfp.rawratio, all.ev$combo_temp, mean)), "_"))[2, ]

temp.plot <- as.data.frame(strsplit(names(tapply(all.ev$gfp.rawratio, all.ev$combo_temp, mean)), "_"))[3, ]

myPalettePurple <- colorRampPalette(rev(brewer.pal(9, "Purples")) )

myPalettePink <- colorRampPalette((brewer.pal(9, "PiYG")) )

#center community fraction heatmaps at white = 0

#create custom color vector with purples and pinks

palette.vec <- rev(c(myPalettePink(11)[1:5], "white", rev(myPalettePurple(9)[1:7]) ))

## Make 3 panel figure

par(mfrow = c(1, 3))

# Create manual range for 32 and 37 color bar, to make them the same

plot.range <- c(.5, 1.31)

# Create vector of range of values at 42 for color bar

range.ratio42 <- as.numeric(range(as.numeric(name.split42$ratio)))

p1 <- ggplot(name.split32, aes(x=as.numeric(gfp.time), y=as.numeric(mch.time))) + geom_tile(aes(fill = as.numeric(ratio)),colour='white') + ggtitle("GFP Ratios 32C") + xlab("GFP Timepoint") + ylab("mCherry Timepoint") + theme_classic() + theme(legend.title = element_blank() ) + scale_x_continuous( breaks = c(1, 5, 9, 13), expand = c(0, 0)) + scale_y_continuous( breaks = c(1, 5, 9, 13), expand = c(0, 0)) + scale_fill_gradientn(colours=rev(palette.vec[5:13]), limits = plot.range) + theme(axis.text=element_text(size=12), axis.title=element_text(size=14) , plot.title = element_text(size=18))

p2 <- ggplot(name.split37, aes(x=as.numeric(gfp.time), y=as.numeric(mch.time))) + geom_tile(aes(fill = as.numeric(ratio)),colour='white') + ggtitle("GFP Ratios 37C") + xlab("GFP Timepoint") + ylab("mCherry Timepoint") + theme_classic() + theme(legend.title = element_blank() ) + scale_x_continuous( breaks = c(1, 5, 9, 13), expand = c(0, 0)) + scale_y_continuous( breaks = c(1, 5, 9, 13), expand = c(0, 0)) + scale_fill_gradientn(colours=rev(palette.vec[5:13]), limits = plot.range) + theme(axis.text=element_text(size=12), axis.title=element_text(size=14) , plot.title = element_text(size=18))

p3 <- ggplot(name.split42, aes(x=as.numeric(gfp.time), y=as.numeric(mch.time))) + geom_tile(aes(fill = as.numeric(ratio)),colour='white') + ggtitle("GFP Ratios 42C") + xlab("GFP Timepoint") + ylab("mCherry Timepoint") + theme_classic() + theme(legend.title = element_blank() ) + scale_x_continuous( breaks = c(1, 5, 9, 13), expand = c(0, 0)) + scale_y_continuous( breaks = c(1, 5, 9, 13), expand = c(0, 0)) + scale_fill_gradientn(colours=rev(palette.vec[c(1,1, 1:8, 11, 13)]), limits = c(range.ratio42[1] , range.ratio42[2] ) ) + theme(axis.text=element_text(size=12), axis.title=element_text(size=14) , plot.title = element_text(size=18))

grid.arrange(p1, p2, p3, nrow = 1)

# Look at whether popualtion size at 42 is related to significant interaction, to evaluate whether abundance could drive an artifact

tapply(all.ev$gfpstd[all.ev$temp == 42], all.ev$lineage[all.ev$temp == 42], mean)

# Lineages with interaction at 42: 1, 2, 7, 10, 11, 13, 15, 18, 19, 20, 21, 22

mean(tapply(all.ev$gfpstd[all.ev$temp == 42], all.ev$lineage[all.ev$temp == 42], mean)[c(1, 2, 7, 10, 11, 13, 15, 18, 19, 20, 21, 22 )])

mean(tapply(all.ev$gfpstd[all.ev$temp == 42], all.ev$lineage[all.ev$temp == 42], mean)[-c(1, 2, 7, 10, 11, 13, 15, 18, 19, 20, 21, 22 )])

sd(tapply(all.ev$gfpstd[all.ev$temp == 42], all.ev$lineage[all.ev$temp == 42], mean)[c(1, 2, 7, 10, 11, 13, 15, 18, 19, 20, 21, 22 )])

sd(tapply(all.ev$gfpstd[all.ev$temp == 42], all.ev$lineage[all.ev$temp == 42], mean)[-c(1, 2, 7, 10, 11, 13, 15, 18, 19, 20, 21, 22 )])

# No significant difference in mean or variance of fraction of population comprised

## Create script to analyze merged growth rate data

library(lme4)

library(KernSmooth)

library(viridis)

library(RColorBrewer)

library(car)

# Make a function to difference the log OD values

log.diff <- function(vector){

diffs <- ((vector[-1])) - (vector[-length(vector)])

}

smooth.fun <- function(vec, width) {

#Find how many values are NA

nas <- sum(is.na(vec))

new.length <- length(vec) - nas

x.vals <- seq(1, new.length)

y.vals <- log(vec[1:new.length] + .02)

smoothed.y <- locpoly(x.vals, y.vals, degree = 1, bandwidth = width, gridsize = new.length)$y

return(smoothed.y)

}

## Read in merged dataset

g.all <- read.csv("~/Desktop/AllGrowthCurves.csv", header = T, row.names = 1)

## Split up merged dataset by temperature and resistance timepoint

c37.all <- g.all[, grepl("C37", colnames(g.all))]

c32.all <- g.all[, grepl("C32", colnames(g.all))]

c42.all <- g.all[, grepl("C42", colnames(g.all))]

# Make smoothed datasets

#### Choose smoothing width

width.all <- 24 # results don't change qualitatively within the range of 12 to 30

c37.mins <- apply(c37.all, 2, min, na.rm = T)

c37.zeroed <- sweep(c37.all, MARGIN = 2, STATS = c37.mins, FUN = "-")

c37.smooth <- apply(c37.zeroed, 2, smooth.fun, width = width.all)

c37.diffs <- lapply(c37.smooth, log.diff)

all.c37.maxdiffs <- unlist(lapply(c37.diffs, max))

c32.mins <- apply(c32.all, 2, min, na.rm = T)

c32.zeroed <- sweep(c32.all, MARGIN = 2, STATS = c32.mins, FUN = "-")

c32.smooth <- apply(c32.zeroed, 2, smooth.fun, width = width.all)

c32.diffs <- lapply(c32.smooth, log.diff)

all.c32.maxdiffs <- unlist(lapply(c32.diffs, max))

c42.mins <- apply(c42.all, 2, min, na.rm = T)

c42.zeroed <- sweep(c42.all, MARGIN = 2, STATS = c42.mins, FUN = "-")

c42.smooth <- apply(c42.zeroed, 2, smooth.fun, width = width.all)

c42.diffs <- lapply(c42.smooth, log.diff)

all.c42.maxdiffs <- unlist(lapply(c42.diffs, max))

## Do same analysis with relative fitness

# Create vectors for anvestral growth rates (at T1)

anc37 <- all.c37.maxdiffs[1:24]

anc32 <- all.c32.maxdiffs[1:24]

anc42 <- all.c42.maxdiffs[1:24]

rel.c37.maxdiffs <- all.c37.maxdiffs / rep(anc37, times = 14)

rel.c32.maxdiffs <- all.c32.maxdiffs / rep(anc32, times = 14)

rel.c42.maxdiffs <- all.c42.maxdiffs / rep(anc42, times = 14)

##Create vector corresponding to resistance timepoint for the above maximum growth rates

res.level <- rep(1:14, each = 24)

## Make plot of average growth rate with std err bars at 3 temperatures, with color changing by resistance level

scale_colour_manual(values = rainbow(8))

par(mfrow = c(1, 2))

plot(0, 0, xlim = c(28, 44), ylim = c(.016, .038), xlab = "Temperature", ylab = "Maximum Growth Rate" , main = "Absolute Growth Rate")

for(tp in 1:14){

gr32 <- mean(all.c32.maxdiffs[res.level == tp])

se32 <- sd(all.c32.maxdiffs[res.level == tp]) / sqrt(24)

gr37 <- mean(all.c37.maxdiffs[res.level == tp])

se37 <- sd(all.c37.maxdiffs[res.level == tp]) / sqrt(24)

gr42 <- mean(all.c42.maxdiffs[res.level == tp])

se42 <- sd(all.c42.maxdiffs[res.level == tp]) / sqrt(24)

points(c(gr32, gr37, gr42) ~ c(32, 37, 42), type = "l", col = rev(viridis(14))[tp], lwd = 2)

points(c(gr32, gr37, gr42) ~ c(32, 37, 42), type = "p", col = rev(viridis(14))[tp], pch = 20)

segments(y0 = c(gr32 + se32/2, gr37 + se37/2, gr42 + se42/2),

y1 = c(gr32 - se32/2, gr37 - se37/2, gr42 - se42/2),

x0 = c(32, 37, 42),

lwd = 3,

col = rev(viridis(14))[tp])

}

legend("topleft", legend = seq(1:14), pch = 20, col = rev(viridis(14)), x.intersp = .5, bty = "n")

plot(0, 0, xlim = c(30, 44), ylim = c(.59, 1.05), xlab = "Temperature", ylab = "Maximum Growth Rate", main = "Relative Growth Rate" )

for(tp in 1:14){

gr.rel32 <- mean(rel.c32.maxdiffs[res.level == tp])

se.rel32 <- sd(rel.c32.maxdiffs[res.level == tp]) / sqrt(24)

gr.rel37 <- mean(rel.c37.maxdiffs[res.level == tp])

se.rel37 <- sd(rel.c37.maxdiffs[res.level == tp]) / sqrt(24)

gr.rel42 <- mean(rel.c42.maxdiffs[res.level == tp])

se.rel42 <- sd(rel.c42.maxdiffs[res.level == tp]) / sqrt(24)

points(c(gr.rel32, gr.rel37, gr.rel42) ~ c(32, 37, 42), type = "l", col = rev(viridis(14))[tp], lwd = 2)

points(c(gr.rel32, gr.rel37, gr.rel42) ~ c(32, 37, 42), type = "p", col = rev(viridis(14))[tp], pch = 20)

segments(y0 = c(gr.rel32 + se.rel32/2, gr.rel37 + se.rel37/2, gr.rel42 + se.rel42/2),

y1 = c(gr.rel32 - se.rel32/2, gr.rel37 - se.rel37/2, gr.rel42 - se.rel42/2),

x0 = c(32, 37, 42),

lwd = 3,

col = rev(viridis(14))[tp])

}

## Format data for regression analysis

## Concatenate all growth rate values into one vector

p1.abs <- c( all.c32.maxdiffs, all.c37.maxdiffs, all.c42.maxdiffs)

## Relative fitess: divide growth rates by respective ancestral growth rates

p1 <- c( rel.c32.maxdiffs, rel.c37.maxdiffs, rel.c42.maxdiffs)

# Create vector for corresponding temps

t1 <- c(rep(c("c32", "c37", "c42"), each = length(p1.abs) / 3) )

# Create vector for corresponding resistance levels

r1 <- rep(rep(1:14, each = 24 ), times = 3 )

# Create vector for lineages

s1 <- rep(1:24, times = length(p1.abs) / 24 )

# Take out any points where strains didn't grow (defined as relative growth rate less than 1% of ancestor)

# The data points with low growth correspond to wells with no change in optical density

p.1 <- p1[p1 > .1]

t.1 <- t1[p1 > .1]

r.1 <- r1[p1 > .1]

s.1 <- as.factor(s1[p1 > .1])

## Count how many strains had increased relative growth in any evolved strain at 37 versus 32 and 42

sum(t1 == "c37" & p1 > 1)

sum(t1 == "c32" & p1 > 1)

sum(t1 == "c42" & p1 > 1)

##############################################################################################################################

# Regression analysis of relative growth rate costs

summary(lmer(p.1 ~ t.1*r.1 + (1|s.1)))

anova(lmer(p.1 ~ t.1*r.1 + (1|s.1)))

summary(lm(p.1 ~ t.1*r.1 + s.1))

anova(lm(p.1 ~ t.1*r.1 + s.1))

## Script to analyze mutation data and merge with competition event data

library(lme4)

mu <- read.csv("~/Desktop/AnnotatedMutationsWithCategories.csv", header = T)

ev <- read.csv("~/Desktop/MergedFlowCytometryDataset.csv", header = T)

# Create a new matrix for this information, which will then be merged into events dataset

lin.vec <- rep(seq(1, 24, 1), each = 4)

time.vec <- rep(c(1, 5, 9, 13), times = 24)

str.vec <- paste(lin.vec, time.vec, sep = "_")

# Create matrix for aggregate mutation numbers

ag <- cbind(lin.vec, time.vec)

ag <- as.data.frame(ag)

rownames(ag) <- str.vec

colnames(ag) <- c("Lineage", "Time")

# Create matching name in mu matric

mu$match.name <- paste(mu$Lineage, mu$Time, sep = "_")

# Create matching name format in events dataframe

ev$gfp.name <- sub('.', '', ev$gfpstrain)

ev$mch.name <- sub('.', '', ev$mchstrain)

# Create a function to find the immediate ancestor of a strain

find.ancestor <- function(strain.name){

lineage <- as.numeric(unlist(strsplit(strain.name, "_"))[1])

timepoint <- as.numeric(unlist(strsplit(strain.name, "_"))[2])

ifelse(timepoint == 1,

name.return <- NA,

name.return <- paste(lineage, time.vec[which(unique(time.vec) == timepoint) - 1], sep = "_")

)

return(name.return)

}

find.ancestor("1_9")

## For counting categories of mutations, create logical column for whether strain has the mutation, and then count them

# Create column for each category of gene

for(cat in unique(mu$GeneName)){

cat.vec <- mu$GeneName == cat

num.cat <- tapply(cat.vec, mu$match.name, sum)

ag[, get("cat")] <- num.cat[match(rownames(ag), names(num.cat))]

ag[, get("cat")][ ! rownames(ag) %in% names(num.cat)] <- 0

}

## Make columns in events dataframe for difference in mutations between competed strains

# Find matching values for each strain, subtract them, and create new column named "DiffIn__"

col.compare <- colnames(ag)[-c(1, 2)]

for(col.create in col.compare){

str1.val <- ag[, get("col.create")][match(ev$gfp.name , rownames(ag))]

str2.val <- ag[, get("col.create")][match(ev$mch.name , rownames(ag))]

new.col.name <- paste0("DiffIn", get("col.create"))

ev[, get("new.col.name")] <- str1.val - str2.val

}

# Relevel to use 37 as the standard so the linear model compares against this temp

ev$temp <- as.factor(ev$temp)

ev$temp <- relevel(ev$temp, ref = "37")

# Remove duplicate columns for genes with same mutation profile

duplicated(as.list(ev))

colnames(ev)[duplicated(as.list(ev))]

ev <- ev[!duplicated(as.list(ev))]

lm.genes <- summary(lm(log(ev$gfp.rawratio) ~

ev$DiffInyahF * ev$temp +

ev$DiffInaraD * ev$temp +

ev$DiffInyaiO * ev$temp +

ev$DiffInacrB * ev$temp +

ev$DiffInacrA * ev$temp +

ev$DiffInacrR * ev$temp +

ev$DiffInacrR.mscK * ev$temp +

ev$DiffInmscK * ev$temp +

ev$DiffInmdfA* ev$temp +

ev$DiffInompF * ev$temp +

ev$DiffInopgH * ev$temp +

ev$DiffInicd.ymfD * ev$temp +

ev$DiffInlomR * ev$temp +

ev$DiffInpaaC * ev$temp +

ev$DiffIndgcF * ev$temp +

ev$DiffInmarR * ev$temp +

ev$DiffInmarA * ev$temp +

ev$DiffInmarB * ev$temp +

ev$DiffInyejA * ev$temp +

ev$DiffInyfaQ.yfaT * ev$temp +

ev$DiffInmenH * ev$temp +

ev$DiffInxdhB * ev$temp +

ev$DiffInagaS * ev$temp +

ev$DiffInelbB.arcZ * ev$temp +

ev$DiffInarcB * ev$temp +

ev$DiffInrplM * ev$temp +

ev$DiffInenvZ * ev$temp +

ev$DiffInompR * ev$temp +

ev$DiffInatpC * ev$temp +

ev$DiffInatpD * ev$temp +

ev$DiffInatpA * ev$temp +

ev$DiffInatpH * ev$temp +

ev$DiffInatpF * ev$temp +

ev$DiffInatpE * ev$temp +

ev$DiffInatpB * ev$temp +

ev$DiffInuvrA * ev$temp +

ev$DiffInuvrA.ssb * ev$temp +

ev$DiffInssb * ev$temp +

ev$DiffInuxuR * ev$temp +

ev$DiffInslt * ev$temp +

ev$DiffIncreB * ev$temp +

ev$lineage * ev$temp ) )

# Convert coefficients into dataframe

gene.coef <- lm.genes$coefficients

lm.genes

## Count significant differences in genes

# Find effect sizes for genes at different temperatures

par(mfrow = c(2, 1))

eff32 <- gene.coef[, 1][grepl("Diff", rownames(gene.coef)) & grepl("temp32", rownames(gene.coef)) ]

eff37 <- gene.coef[, 1][grepl("Diff", rownames(gene.coef)) & !grepl("temp32", rownames(gene.coef)) & !grepl("temp42", rownames(gene.coef)) ]

eff42 <- gene.coef[, 1][grepl("Diff", rownames(gene.coef)) & grepl("temp42", rownames(gene.coef)) ]

par(mfrow = c(3, 1))

hist(eff37 + eff32, breaks = 10, xlim = c(-2.5, 2.5), ylim = c(0, 10), main = "Estimated Gene Effects at 32 Degrees", xlab = "Effect Size")

#abline(v = median(eff37 + eff32))

hist(eff37 , breaks = 10, xlim = c(-2.5, 2.5), ylim = c(0, 10), main = "Estimated Gene Effects at 37 Degrees", xlab = "Effect Size")

#abline(v = median(eff37 ))

hist(eff37 + eff42, breaks = 15, xlim = c(-2.5, 2.5),ylim = c(0, 10), main = "Estimated Gene Effects at 42 Degrees", xlab = "Effect Size")

#abline(v = median(eff37 + eff42))

effect.32 <- eff37 + eff32

effect.42 <- eff37 + eff42

var.test(effect.32, eff37, alternative = "two.sided")

var.test(effect.42, eff37, alternative = "two.sided")

####

#### Make barchart for mutations at different time points and analyze distribution of categories over time

## Make a plot of new mutations arising at different time points

new.mu <- mu[mu$Time == 1, ]

for(str in unique(mu$match.name)){

if(unlist(strsplit(str, "_"))[2] > 1){

anc <- find.ancestor(str)

str.mut <- mu[mu$match.name == str, ]

anc.mut <- mu[mu$match.name == anc, ]

new.mu <- rbind(new.mu, str.mut[!str.mut$Location %in% anc.mut$Location, ])

}

}

# Count mutations in each category at each time point

tapply(new.mu$Category2[new.mu$Time == 1], new.mu$Category2[new.mu$Time == 1], length)

tapply(new.mu$Category2[new.mu$Time == 5], new.mu$Category2[new.mu$Time == 5], length)

tapply(new.mu$Category2[new.mu$Time == 9], new.mu$Category2[new.mu$Time == 9], length)

tapply(new.mu$Category2[new.mu$Time == 13], new.mu$Category2[new.mu$Time == 13], length)

# Create dataset for barchart

new.counts <- c(

tapply(new.mu$Category2[new.mu$Time == 1], new.mu$Category2[new.mu$Time == 1], length),

tapply(new.mu$Category2[new.mu$Time == 5], new.mu$Category2[new.mu$Time == 5], length),

tapply(new.mu$Category2[new.mu$Time == 9], new.mu$Category2[new.mu$Time == 9], length),

tapply(new.mu$Category2[new.mu$Time == 13], new.mu$Category2[new.mu$Time == 13], length)

)

#Create times for barchart

chart.times <- as.factor(c(

rep(1, times = length(tapply(new.mu$Category2[new.mu$Time == 1], new.mu$Category2[new.mu$Time == 1], length) ) ),

rep(5, times = length(tapply(new.mu$Category2[new.mu$Time == 5], new.mu$Category2[new.mu$Time == 5], length) ) ),

rep(9, times = length(tapply(new.mu$Category2[new.mu$Time == 9], new.mu$Category2[new.mu$Time == 9], length) ) ),

rep(13, times = length(tapply(new.mu$Category2[new.mu$Time == 13], new.mu$Category2[new.mu$Time == 13], length) ) )

) )

Category <- names(new.counts)

bar.chart <- data.frame(new.counts, Category, chart.times)

## Re-order bar chart to have colors go by mean appearance time

bar.chart$time.mult <- bar.chart$new.counts * as.numeric(bar.chart$chart.times)

mean.times <- tapply(bar.chart$time.mult, bar.chart$Category, sum) / tapply(bar.chart$new.counts, bar.chart$Category, sum)

bar.chart$Category <- factor(bar.chart$Category, levels = rev(names(sort(mean.times)) ) )

ggplot(bar.chart, aes(fill=Category, y=new.counts, x=chart.times)) +

geom_bar(position="stack", stat="identity") +

xlab("Timepoint") +

ylab("Number of New Mutations")

## Now, chi-squared tests of distribution of mutations over time

# Evaluate which categories are unevenly distributed among timepoints

chi.sq <- vector()

for(cat2 in unique(bar.chart$Category)){

place <- which(cat2 == unique(bar.chart$Category))

chi.sq[place] <- (chisq.test(bar.chart$new.counts[bar.chart$Category == cat2]))$p.value

}

names(chi.sq) <- unique(bar.chart$Category)

# Count number of mutations at different times and in different categories

tapply(mu$Time, mu$Time, length)

tapply(mu$Category2, mu$Category2, length)

tapply(new.mu$Time, new.mu$Time, length)

## Check whether any specific mutation category is associated with the presence of greater cost at 42 degrees

# Lineages with interaction at 42: 1, 2, 7, 10, 11, 13, 15, 18, 19, 20, 21, 22

# Create matrix for presence or absence of each mutation category in each lineage

lin.cat <- vector()

for(c1 in unique(mu$Category2)){

lin.tf <- as.numeric(seq(1:24) %in% unique(mu$Lineage[mu$Category2 == c1]))

lin.cat <- rbind(lin.cat, lin.tf)

}

rownames(lin.cat) <- unique(mu$Category2)

colnames(lin.cat) <- 1:24

# Do Fisher's exact test on lineages with interactions and mutation category

int.lins <- c(1, 1, 0, 0, 0, 0, 1, 0, 0, 1, 1, 0, 1, 0, 1, 0, 0, 1, 1, 1, 1, 1, 0, 0)

names(int.lins) <- seq(1:24)

apply(lin.cat, 1, fisher.test, y = int.lins, simulate.p.value = T)

# Calculate probability of finding 5 significant categories by chance

# Equals 10 choose 5 (252) * .05^5 * .95^5

252 * (.05^5) * .95^5
